# Alkali-Activated Granulated Aggregates from Low-Quality Fly Ash and Basalt Dust: Effect of Sodium Silicate/NaOH Activator Chemistry and Accelerated Carbonation

**DOI:** 10.3390/ma19102026

**Published:** 2026-05-13

**Authors:** Krzysztof Granatyr, Małgorzata Franus, Katarzyna Kalinowska-Wichrowska, Adam Masłoń

**Affiliations:** 1 Faculty of Civil Engineering and Environmental Sciences, Bialystok University of Technology, Wiejska 45E, 15-351 Bialystok, Poland; krzysztof.granatyr@pb.edu.pl (K.G.); k.kalinowska@pb.edu.pl (K.K.-W.); 2 Department of General Construction, Lublin University of Technology, Nadbystrzycka 40 Street, 20-618 Lublin, Poland; m.franus@pollub.pl; 3 Department of Environmental Engineering and Chemistry, Rzeszow University of Technology, Powstańców Warszawy 12 Av., 35-029 Rzeszów, Poland

**Keywords:** mineral waste recycling, alkali-activated aggregates, low-carbon aggregates, biomass fly ash, coal fly ash, basalt dust, carbonation hardening, CO_2_ sequestration from minerals, granulated aggregates, circular economy

## Abstract

This study examined alkali-activated granular aggregates produced from biomass fly ash, coal fly ash, and basalt dust. The work focused on multicomponent industrial waste mixtures activated with two sodium silicate-based systems and on the effect of carbonation curing on aggregate properties. Twelve designed mixtures and reference series were evaluated in terms of particle density, water absorption, and mechanical performance. The response to carbonation was also analysed to assess the potential for CO_2_ uptake. Mechanical performance ranged from low to moderate and depended on mixture composition, activator type, and carbonation treatment. In most cases, the blended activator produced higher strength before carbonation than sodium silicate alone, whereas carbonation frequently reduced strength. Mixtures containing more basalt dust and less biomass fly ash generally showed the most favourable combination of properties. The results indicate that these industrial mineral wastes can be used to produce alkali-activated granular aggregates with adjustable properties, while carbonation curing may additionally contribute to phase changes and limited CO_2_ binding.

## 1. Introduction

Decarbonization of the construction industry remains a major challenge because the sector depends both on high-emission cementitious binders and on very large quantities of mineral aggregates [1,2]. In this context, artificial aggregates produced from waste are increasingly considered a promising circular economy approach, as they enable the conversion of fine industrial residues into granular materials suitable for mortars and concrete [2,3]. Compared with sintered lightweight aggregates, cold-bonded and alkali-activated routes are attractive because they require less thermal energy and allow the use of industrial by-products [3,4]. However, these systems still show important limitations, especially in terms of mechanical strength, water absorption, and durability, which restrict their wider structural use [4,5].

Alkali-activated materials (AAMs) are relevant here because they enable the hardening of fine powders at ambient or moderately elevated temperatures [4,6]. Their carbonation behaviour, however, is complex and strongly dependent on the precursor composition, activator chemistry, and curing conditions. Depending on the system, carbonation may promote densification and lower water absorption, but it may also cause unfavourable microstructural changes and reduce mechanical performance [6,7,8]. Recent studies indicate that durability, activator efficiency, and practical scalability remain the main barriers to broader implementation of alkali-activated technology [9,10].

An additional issue is the variability of waste precursors. Biomass fly ash and coal fly ash can differ markedly in mineralogy, chemical composition, loss on ignition, and reactivity, and therefore cannot be treated as directly interchangeable in alkali-activated systems [11,12]. Basalt dust may also influence the performance of such systems through its effect on particle packing, reactivity, and interaction with the binder matrix [13]. This is particularly significant in the case of granular aggregates, where particle packing, particle dissolution, and interparticle bonds determine density, water absorption, and resistance to crushing. Previous studies have shown that alkali-activated or geopolymer granulated aggregates can be produced from fly ash, slag, basalt-containing mixtures, and other industrial residues [14,15]. Carbonation curing has also been reported to improve selected aggregate properties, although the effect depends strongly on the precursor composition and curing regime [16,17]. More recent work on geopolymer granules and carbonated alkali-activated aggregates suggests that composition optimisation is essential if waste-derived aggregates are to achieve acceptable technical performance [18,19]. However, most published studies still focus on systems based on a single precursor, a single activator formulation, or a single curing route. As a result, the behaviour of multicomponent waste mixtures containing biomass fly ash, coal fly ash with different LOIs, and basalt dust under different sodium silicate-based activation conditions remains insufficiently described.

The aim of this study was therefore to produce alkali-activated granular aggregates from biomass fly ash, coal fly ash with contrasting LOIs, and basalt dust, and to determine how mixture composition and activator type affect their key engineering properties and response to accelerated carbonation. Two activation methods based on the use of sodium silicate were compared. The aggregates were assessed before and after carbonation to determine the composition ranges that ensure beneficial performance characteristics.

## 2. Materials and Methods

### 2.1. Materials

Sodium silicate solution (water glass) and sodium hydroxide were used as alkaline activators to form a binding phase responsible for the consolidation of the granules. Two activation methods were investigated: (i) sodium silicate solution used as the sole activator, and (ii) a mixed activator consisting of sodium silicate solution and 12 M NaOH in a mass ratio of 2.5:1, introduced to increase alkalinity and improve the solubility of the precursor. The main parameters of the sodium silicate and NaOH solutions used in the study are given in Table 1.

### 2.2. Research Methodology

#### 2.2.1. Research Plan

A mixture design comprising 12 formulations (series 1–12) was adopted, as presented in Table 2. In this design, the proportions of three constituents were varied: biomass fly ash (CHP plant in Białystok, Poland, fluidized bed boiler), basalt dust (Trzuskawica S.A. Targowica, Poland), and fly ash (high-LOI) (CHP plant in Białystok, Poland, pulverised coal boiler), while the contribution of Fly Ash A (CFPP in Ostrołęka, Poland, pulverised coal boiler) was kept constant at 5% in all mixes. In addition, reference series were prepared using each raw material individually (100% of a given precursor). The alkaline activator was dosed at a constant activator to solids ratio of 1:3 (by mass; 1 part activator to 3 parts dry solids) in two variants: (i) sodium silicate solution (water glass) and (ii) a blended activator composed of sodium silicate solution and 12 M NaOH, mixed at a mass ratio of 2.5:1 (sodium silicate solution to 12 M NaOH).

#### 2.2.2. Method

Particle size distribution

The particle size distribution of the manufactured aggregates was assessed using three replicate samples in accordance with PN-EN 12620+A1:2010 [20]. Particle density and water absorption were determined in accordance PN-EN 1097-6:2022-07 [21].

Single aggregate crushing strength test

The mechanical resistance of the granules was evaluated using a single granule crushing test, in which each specimen was placed between two parallel plates and loaded to failure. The tests were carried out using an Inspect Table universal testing machine (50 kN; Hegewald und Peschke MPT GmbH, Nossen, Germany). The upper plate was displaced at 2 mm·min^−1^. Before testing, the external diameter of each granule was measured in two orthogonal directions, and the mean value d was used in the calculations; granule height was recorded only as an auxiliary geometrical parameter. Loading continued until the recorded force decreased by 30% from the peak value. Owing to the limited elastic deformation of the granules, all specimens fractured during the test. At least eight granules were tested for each processing condition, and the maximum failure load F was recorded. On this basis, the single aggregate crushing strength test S was calculated using Equation (1) as a comparative mechanical index [22,23,24,25,26,27,28,29]:

(1) $$S=\frac{2.8F}{\pi d^{2}}$$

where F is the maximum failure load, and d is the mean external granule diameter. Because the tested granules were approximately spherical rather than geometrically regular specimens, the obtained value should be treated as a comparative mechanical parameter rather than as conventional compressive strength.

**Figure 1:**
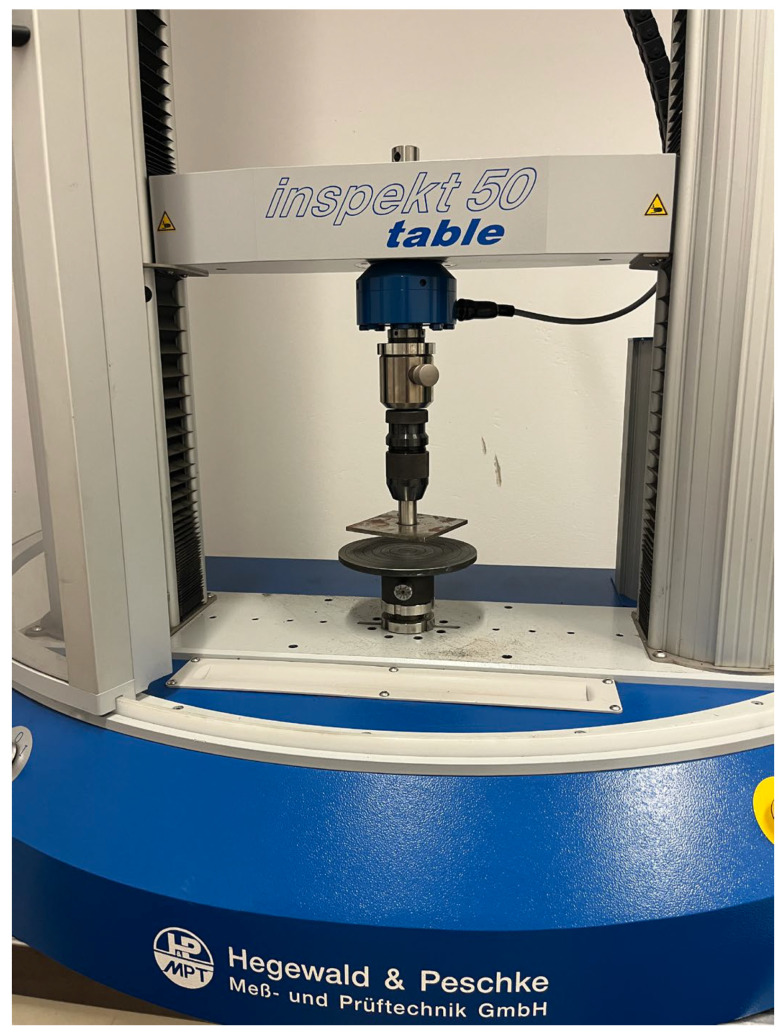
Single aggregate crushing strength testing machine.

Figure 1 presents the test setup used to determine the single aggregate crushing strength test.

Water absorption was measured in accordance with PN-EN 1097-6:2022-07 [21] using eight replicate samples. The WA value was calculated according to Equation (2).


(2) $$WA,\%=\frac{m_{2}-m_{1}}{m_{1}}\cdot 100\%$$

Chemical composition

Chemical composition of the raw materials was determined by X-ray fluorescence (XRF) using a ZSX PRIMUS IV spectrometer (Rigaku, Tokyo, Japan) equipped with a 4 kW power source. Phase composition was characterised by X-ray diffraction (XRD) using a Bruker D8 Discover A25 diffractometer (Bruker, Karlsruhe, Germany) with CuKα radiation (λ = 1.54050 Å), operated at 40 kV and 30 mA. Diffraction data were collected over a 2θ range of 10–70°, with a step size of 0.006° (2θ) and a scanning rate of 0.006° (2θ)·min^−1^.

DTA/TGA

Differential thermal analysis (DTA) and thermogravimetric analysis (TGA) were performed using a Netzsch STA 409 PG analyser (NETZSCH-Gerätebau GmbH, Selb, Germany), recorded in air. Samples were heated to 1100 °C at 10 °C·min^−1^.

Optical Microscopy and Scanning Microscopy Analysis.

Themorphology of the artificial aggregates was examined by scanning electron microscopy (SEM) using a high-resolution Tescan microscope and an FEI Quanta 250 FEG microscope FEI Company, Hillsboro, OR, USA with a thermally assisted field emission source (Schottky Emitter). Optical microscopy observations were carried out using an Opta-Tech microscope (Opta-Tech Sp. z o.o., Warsaw, Poland) equipped with a CCD camera.

Carbon footprint analysis

The carbon footprint assessment was carried out in accordance with PN-EN ISO 14067:2018-10 [30], while the detailed calculation assumptions were defined for the studied system.

### 2.3. Production Process of Artificial Aggregate

**Figure 2:**
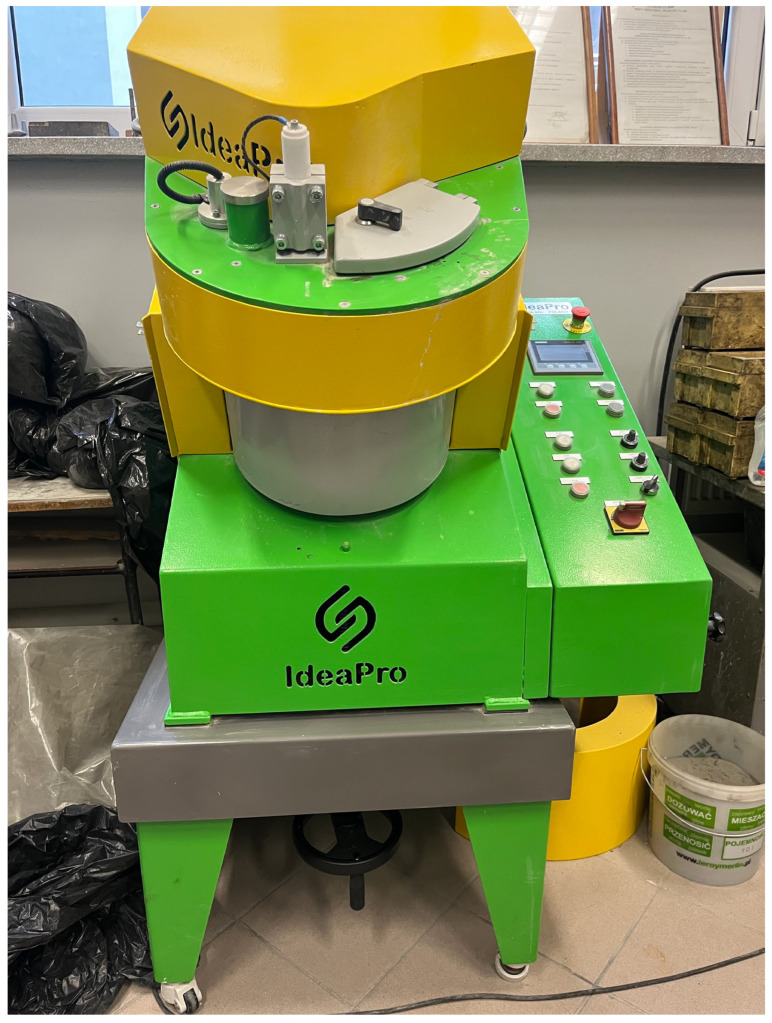
The machine used for artificial aggregate production.

The production of the artificial aggregate began by weighing the required amounts of each constituent together with the activator and water, according to the experimental design. The dry materials were mixed for 3 min and then transferred to the granulator. The activator water solution was subsequently sprayed in gradually during granulation. In Figure 2, the granulation machine used for aggregate production is shown.

The produced granulated aggregates were first cured for 14 days under laboratory conditions and subsequently exposed to accelerated carbonation for 13 days in a chamber operated at 10% CO_2_, 95% relative humidity, and 25 °C. After removal from the chamber, the aggregates were air-dried for 24 h and tested after 28 days.

## 3. Results and Discussion

### 3.1. Raw Material Characterisation (PSD, XRF, XRD, TG/DTG/DTA)

**Figure 3:**
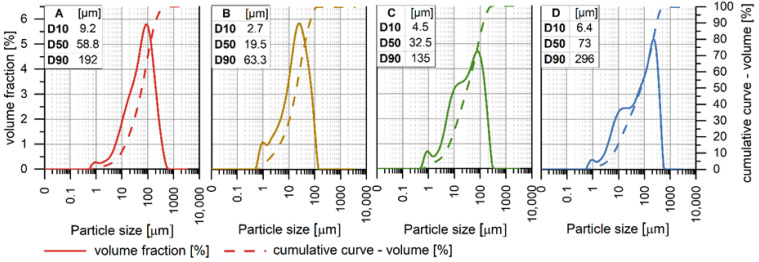
Particle size distribution (PSD) raw materials ((**A-red**)—fly ash—high-LOI, (**B-orange**)—basalt dust, (**C-green**)—Fly Ash A, and (**D-blue**)—biomass fly ash).

The particle size distribution and cumulative volume curves of the tested raw materials are presented in Figure 3. Fly ash with a high LOI (A) is characterised by a moderately fine and relatively broad particle size distribution, with D10 = 9.2 µm, D50 = 58.8 µm, and D90 = 192 µm. Basalt dust (B) is the finest precursor in the tested set, with D10 = 2.7 µm, D50 = 19.5 µm, and D90 = 63.3 µm. Fly Ash A (C) exhibits a fine-grained particle size distribution, intermediate between basalt dust and high-LOI fly ash, with D10 = 4.5 µm, D50 = 32.5 µm, and D90 = 135 µm. In contrast, biomass fly ash (D) is the coarsest material, with D10 = 6.4 µm, D50 = 73 µm, and D90 = 296 µm.

The particle size parameters of the two coal fly ashes investigated in this study fall within the range of variability reported in the literature for hard coal fly ashes from pulverised fuel boilers [31]. Fly Ash A exhibited a finer particle size distribution than the high-LOI fly ash. The particle size distribution of the basalt dust used in this study was comparable to that reported in the literature for basalt powder [32].

**Figure 4:**
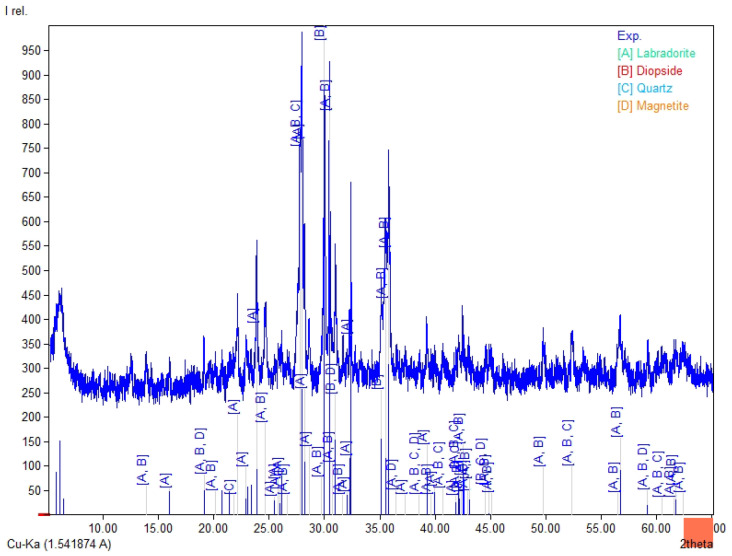
XRD pattern for basalt dust, A—labradorite ((Ca,Na)(Al,SI)_4_O_8_) B—diopside (CaMgSi_2_O_6_), C—quartz (SiO_2_), and D—magnetite (Fe_3_O_4_).

**Figure 5:**
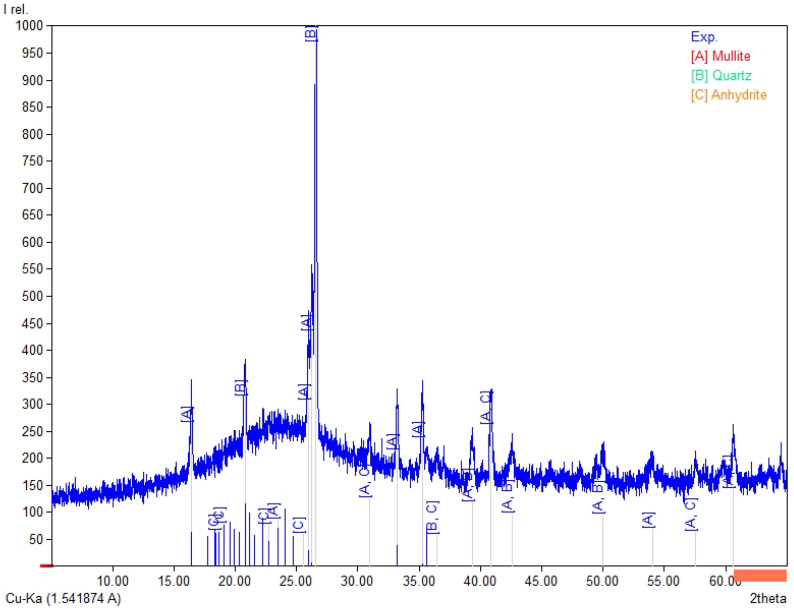
XRD pattern for fly ash—high-LOI, A—mullite (Al_6_Si_2_O_13_), B—quartz (SiO_2_), and C—anhydrite (CaSO_4_).

**Figure 6:**
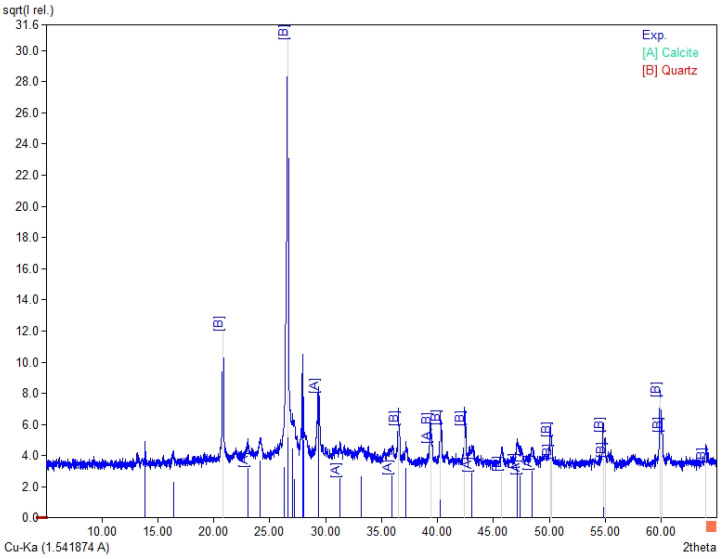
XRD pattern for biomass fly ash, A—calcite (CaCO_3_) and B—quartz (SiO_2_).

**Figure 7:**
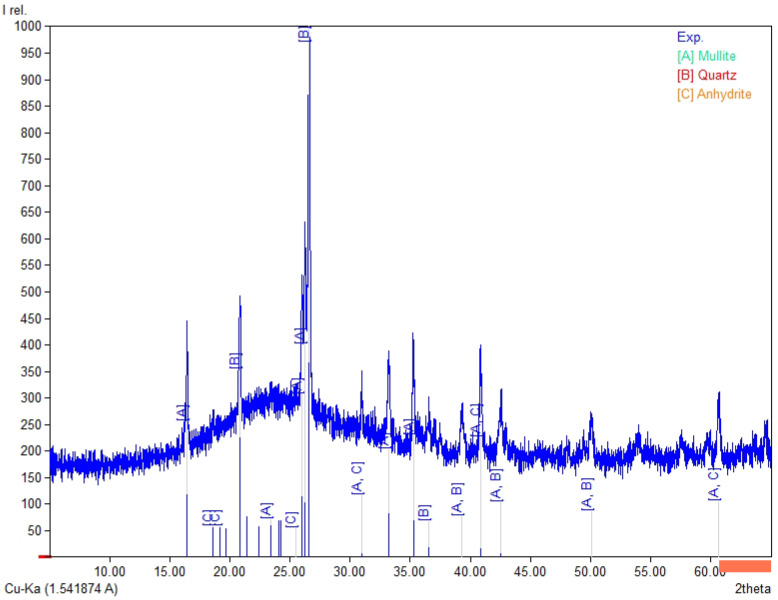
XRD pattern for Fly Ash A, A–mullite (Al_6_Si_2_O_13_), B—quartz (SiO_2_), and C—anhydrite (CaSO_4_).

The chemical composition (Table 3) of the tested fly ashes and basalt dust reveals clear differences between the raw materials. Fly Ash A and the high-LOI fly ash are rich in SiO_2_ (35.5–38.6 wt.%) and Al_2_O_3_ (15.1–16.1 wt.%). In both materials, the combined SiO_2_ + Al_2_O_3_ + Fe_2_O_3_ content is similar (56.7–60.2 wt.%), whereas CaO remains low (about 1.9–2.1 wt.%). Biomass fly ash differs clearly from the coal fly ashes, mainly because of its higher CaO content (16.0 wt.%) and lower SiO_2_ and Al_2_O_3_ contents (29.7 and 7.55 wt.%, respectively), which results in a lower SiO_2_ + Al_2_O_3_ + Fe_2_O_3_ sum (41.1 wt.%). It also contains higher SO_3_ (2.32 wt.%) and detectable chloride (0.21 wt.%). Basalt dust contains more Fe_2_O_3_ (12.8 wt.%), CaO (8.74 wt.%), MgO (5.92 wt.%), and TiO_2_ (2.53 wt.%) and shows the lowest LOI (3.58 wt.%). The LOI values vary considerably among the tested materials and are highest for the high-LOI fly ash (10.8 wt.%), followed by biomass fly ash (9.1 wt.%) and Fly Ash A (4.5 wt.%). These differences may affect water demand and the behaviour of the binder–activator system. In Figure 4, Figure 5, Figure 6 and Figure 7, the XRD results for basalt dust, fly ash type high-LOI, biomass fly ash and Fly Ash A are shown.

The XRD pattern of basalt dust (Figure 4) is dominated by reflections assigned to labradorite (A; (Ca,Na)(Al,Si)_4_O_8_) and diopside (B; CaMgSi_2_O_6_), with minor quartz (C; SiO_2_) and accessory magnetite (D; Fe_3_O_4_). This phase composition is consistent with basalt-derived mineral powder reported in the literature [31].

The XRD pattern of the high-LOI fly ash (Figure 5) is dominated by mullite (A; Al_6_Si_2_O_13_) and quartz (B; SiO_2_), with a minor amount of anhydrite (C; CaSO_4_). This phase composition is typical of coal-derived fly ashes, in which quartz and mullite usually represent the main crystalline phases, whereas sulfate phases may occur in smaller amounts depending on fuel composition and combustion conditions [31].

Biomass fly ash (Figure 6) is characterised by a phase composition dominated by calcite (A; CaCO_3_), while quartz (B; SiO_2_) occurs as a secondary crystalline phase. This phase composition is a characteristic feature of biomass fly ash, in which calcite and quartz constitute residues derived from the sludge of a fluidised-bed furnace [33].

Fly Ash A (Figure 7) contains mullite (A; Al_6_Si_2_O_13_) and quartz (B; SiO_2_) as the main crystalline phases, with a minor amount of anhydrite (C; CaSO_4_). This phase composition is typical of coal-derived fly ash, in which anhydrite is a residue from the flue gas desulfurization process [31].

**Figure 8:**
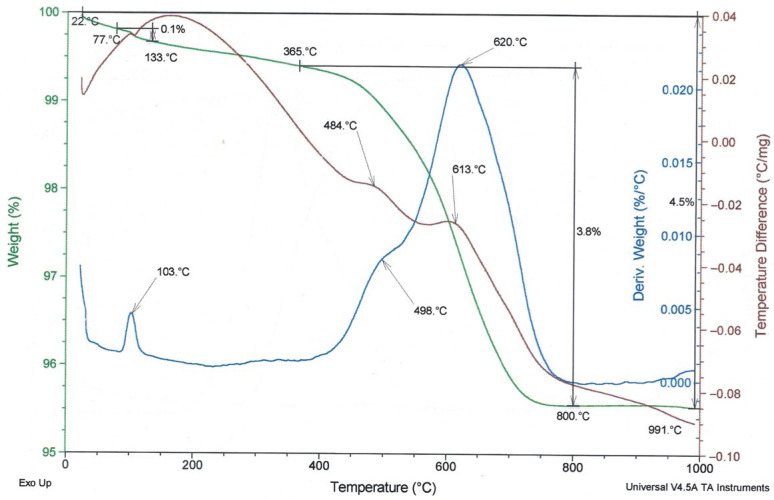
TG/DTG and DTA curves of Fly Ash A.

**Figure 9:**
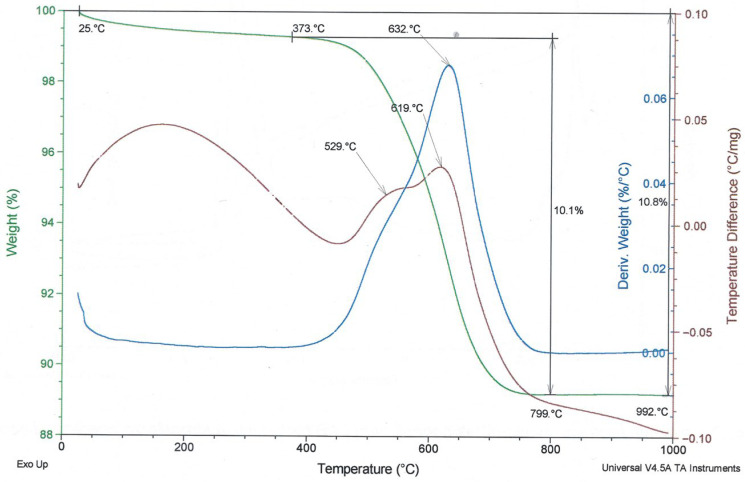
TG/DTG and DTA curves of fly ash—high-LOI.

**Figure 10:**
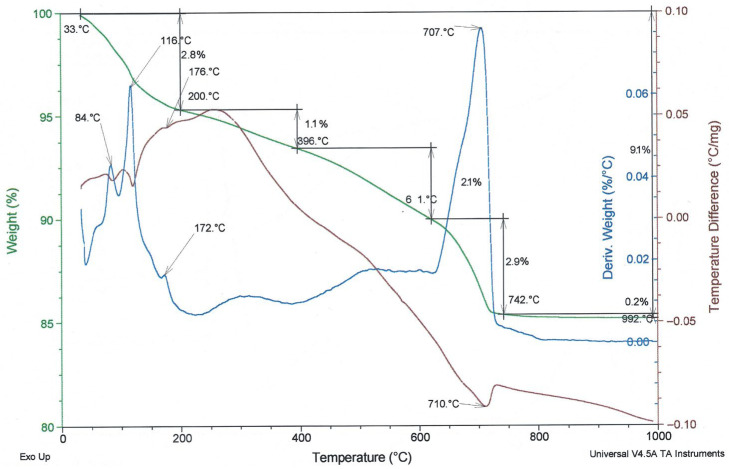
TG/DTG and DTA curves of biomass fly ash.

To compare the thermal behaviour of the examined fly ashes, TG/DTG and DTA curves are presented in Figure 8, Figure 9 and Figure 10. In these figures, the green line represents the TG/weight change curve, the blue line represents the DTG/derivative weight curve, and the brown line represents the DTA/temperature difference curve; the black guide lines indicate selected mass-loss ranges and corresponding percentage changes.

Both analysed fly ashes (high-LOI fly ash and type A fly ash) exhibit similar thermal curves, indicating a similar material nature and transformations occurring within a comparable temperature range. The difference consists of the higher loss on ignition of fly ash with high LOI (Figure 9) than Fly Ash A (Figure 8), due to the higher unburned carbon content. It should also be noted that the mullite, anhydrite, and quartz phases present in both ashes cannot be clearly identified in the DTA diagrams because their characteristic transformations occur outside the temperature range used in the study or produce too weak thermal effects.

The TG/DTG/DTA curves of the biomass fly ash (Figure 10) are consistent with the typical thermal behaviour reported for biomass fly ash [33], especially for materials with an increased content of calcium-bearing phases. This is indicated by the relatively high mass loss and the distinct thermal effect observed in the range of approximately 700–750 °C, which can be attributed to calcite decomposition.

The conducted studies revealed significant differences among the analysed raw materials in terms of particle size distribution, chemical and phase composition, and thermal properties. The XRD and thermal analysis results complement each other and indicate that these raw materials may be promising precursors for alkali-activated materials due to the presence of reactive aluminosilicate components and calcium-bearing phases. Furthermore, the calcium-rich components may provide a basis for CO_2_ sequestration through carbonation.

### 3.2. Single Aggregate Crushing Strength Test

**Figure 11:**
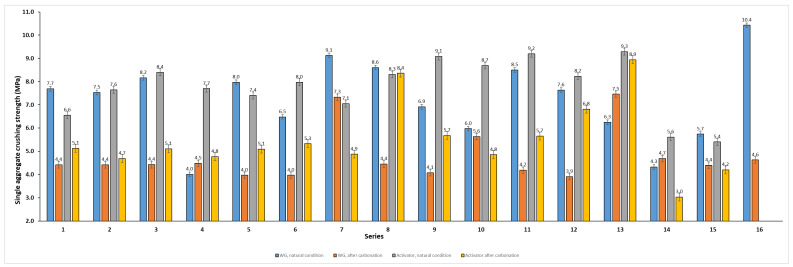
Single aggregate crushing strength of the investigated mixtures (series 1–12) and single material reference series (FA A-100, FA (high-LOI)-100, Bio-100, and Basalt-100; series 13–16) produced using two activator systems (water glass and blended Activator 2.5) and tested under natural conditions and after carbonation curing. Error bars represent the standard error of the mean (SE).

Before carbonation, both systems reached a similar strength level, although mixtures activated with Activator 2.5 more frequently yielded higher, less scattered results (Figure 11). In the water glass system, carbonation reduced single aggregate crushing strength in most series, and only a few mixtures showed an improvement. The highest pre-carbonation strength in the water glass series was observed for series 16 (10.4 MPa), while after carbonation, the highest value was recorded for series 13 (7.5 MPa). In series 8, where the ratio of Na_2_SiO_3_/NaOH = 2.5, carbonation also reduced strength in most cases, but the losses were usually smaller than for water glass. The highest strength before carbonation was obtained for series 13 (9.3 MPa), which also remained the strongest after carbonation (8.9 MPa). Series 8 was particularly notable, as its single aggregate crushing strength remained almost unchanged after carbonation (8.3–8.4 MPa). These results indicate that in series 8, where the ratio of Na_2_SiO_3_/NaOH = 2.5 provided better strength retention after carbonation than water glass. The results were greater than those obtained by researchers using other precursors [34]. Although the chemical composition of the precursor used in series 13 (Fly Ash A) was related to (FA (high-LOI)) (Series 14), this behaviour can be tentatively attributed to the high unburned carbon content in the fly ash with high LOI. Due to the developed surface area, the unburned carbon could influence the binding process and help form a sufficiently stable structure [35].

**Figure 12:**
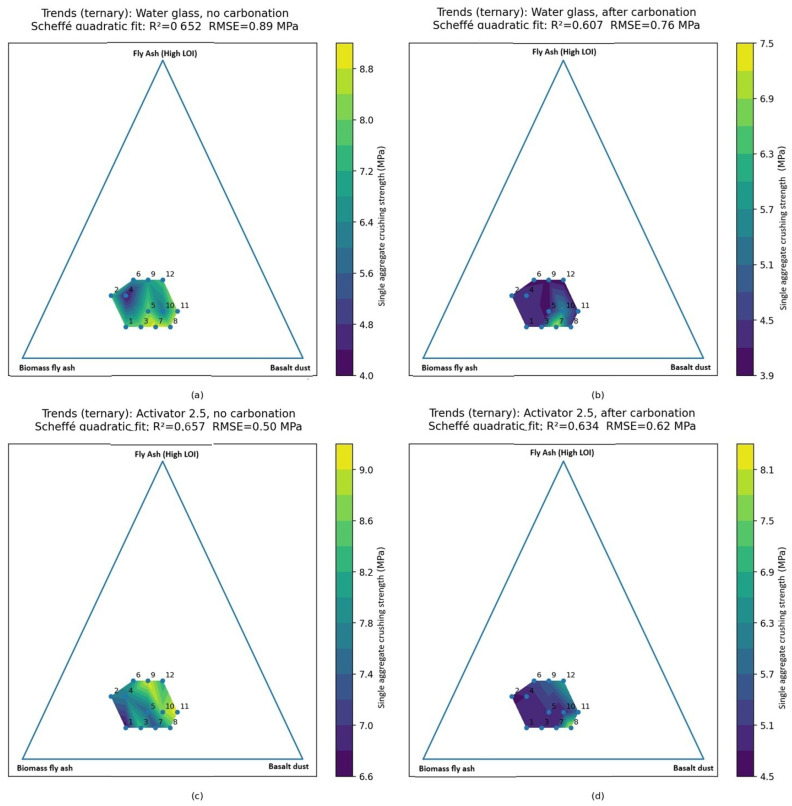
Ternary response–surface plots of single aggregate crushing strength predicted by a Scheffé quadratic mixture model within the constrained design space (Fly Ash A determined at 5 wt.%). (**a**) Water glass, no carbonation; (**b**) water glass, after carbonation; (**c**) Activator 2.5, no carbonation; and (**d**) Activator 2.5, after carbonation.

**Figure 13:**
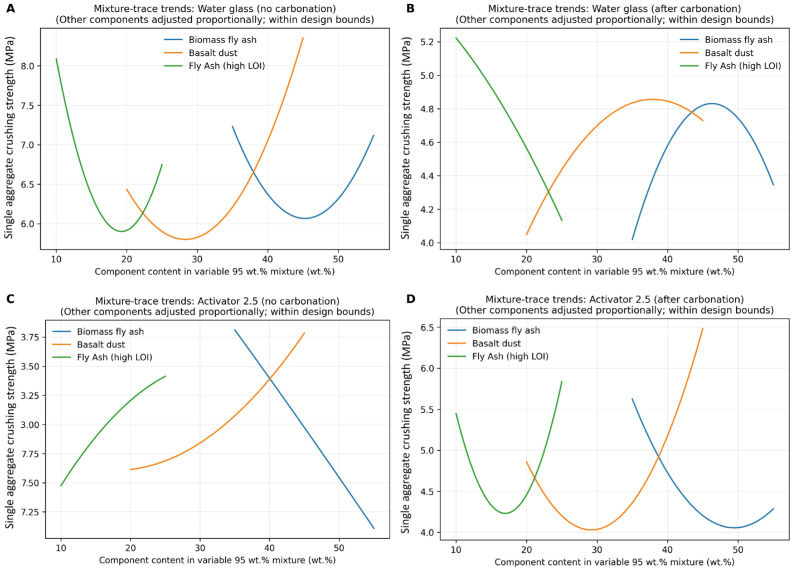
Mixture–trace plots showing the predicted effect of varying each component within the constrained mixture design space (Fly Ash A determined at 5 wt.%). The response is single aggregate crushing strength (MPa) estimated from a second-order Scheffé mixture model; when one component is varied, the remaining components are adjusted proportionally to maintain the constant total of the variable 95 wt.% fraction. (**A**) Water glass, no carbonation; (**B**) water glass, after carbonation; (**C**) Activator 2.5, no carbonation; and (**D**) Activator 2.5, after carbonation.

The relationships between composition and strength are further illustrated by the ternary response–surface and mixture–trace plots (Figure 12 and Figure 13). The fitted Scheffé quadratic models reproduced the main compositional trends, although their predictive accuracy remained moderate (R^2^ ≈ 0.61–0.66; RMSE ≈ 0.50–0.89 MPa). For this reason, the models are better suited to show general tendencies than to provide precise predictions. Both types of plots indicate that increasing the basalt powder content generally improves single aggregate crushing strength, whereas increasing the biomass fly ash content tends to reduce it, particularly in the Activator 2.5. The role of the high-LOI fly ash was less straightforward and depended on the activator system. After carbonation, the predicted response shifted toward lower strength values, which suggests that the effect of carbonation depended on mixture composition and was not uniform across all blends. The obtained strength levels are consistent with the broader range reported for cold-bonded and alkali-activated aggregates and approach the lower to intermediate mechanical range reported for selected commercial lightweight aggregates [36].

The strength variations could be partly explained by the different chemical compositions of the precursors. Fly Ash A and the high-LOI fly ash were the richest in SiO_2_ and Al_2_O_3_, which made them the main potential sources of aluminosilicate species for alkali activation. However, the high LOI of the latter may indicate a higher content of unburned carbon and other thermally removable components, which could increase liquid demand and reduce the effective availability of the alkaline activator. Biomass fly ash was rich in CaO, but its lower SiO_2_ and Al_2_O_3_ contents may have limited its contribution to the formation of a continuous aluminosilicate matrix. Basalt dust, despite being less alumina-rich than the coal fly ashes, had low LOI and relatively high SiO_2_, CaO, MgO, and Fe_2_O_3_ contents, which may have supported better packing and the formation of a more compact granular structure. This interpretation is consistent with the favourable performance of basalt-rich and low high-LOI mixtures, particularly series 8.

### 3.3. Particle Density

**Figure 14:**
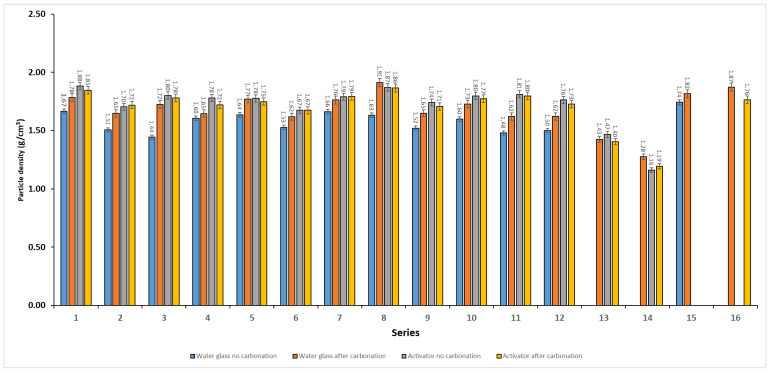
Particle density of the investigated mixtures (series 1–12) and single-material reference series (FA A-100, FA (high-LOI)-100, Bio-100, and Basalt-100) produced using two activator systems (water glass and blended Activator 2.5) and tested before and after carbonation curing. Error bars represent the standard error of the mean (SE).

**Figure 15:**
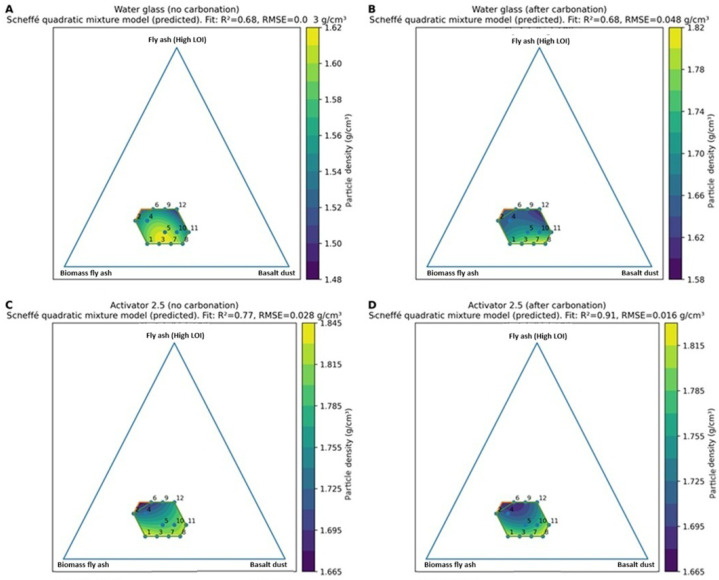
Ternary response–surface plots of particle density predicted by a Scheffé quadratic mixture model within the constrained design space (Fly Ash A determined at 5 wt.%). (**A**) Water glass, no carbonation; (**B**) water glass, after carbonation; (**C**) Activator 2.5, no carbonation; and (**D**) Activator 2.5, after carbonation.

**Figure 16:**
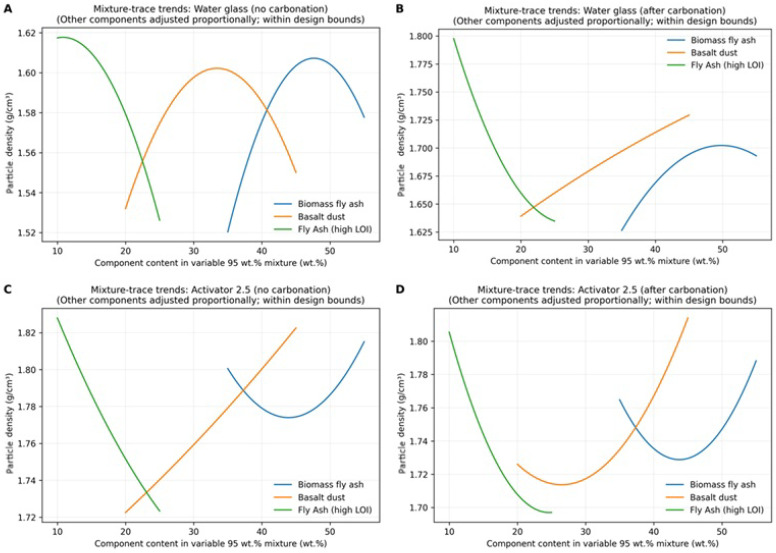
Mixture–trace plots showing the predicted effect of varying each component within the constrained mixture design space (Fly Ash A determined at 5 wt.%). The response is particle density estimated from a second-order Scheffé mixture model; when one component is varied, the remaining components are adjusted proportionally to maintain the constant total of the variable 95 wt.% fraction. (**A**) Water glass, no carbonation; (**B**) Water glass, after carbonation; (**C**) Activator 2.5, no carbonation; (**D**) Activator 2.5, after carbonation.

Particle density results for the designed blends (series 1–12) are presented in Figure 14 for both activator systems and curing conditions. Under natural conditions, the Activator 2.5 gave higher particle density than the water glass system (mean 1.782 vs. 1.565 kg/dm^3^), indicating a denser structure. After carbonation, particle density increased in all water glass mixtures, with the highest value of 1.91 kg/dm^3^ recorded for series 8. In contrast, the effect of carbonation in series 8, where the ratio of Na_2_SiO_3_/NaOH = 2.5, was small. Based on the measured particle density values, the produced aggregates can be classified as lightweight aggregates in accordance with EN 13055, since their particle density remained below 2000 kg/m^3^ [5]. For the series in which no results are presented, the specimens underwent degradation during conditioning in water, which prevented further testing. The composition–density relationships were described using Scheffé quadratic mixture models and are shown as ternary response–surface (Figure 15) and mixture–trace plots (Figure 16). The models showed moderate to very good agreement with the experimental data (R^2^ ≈ 0.68–0.91). Increasing the proportion of high-LOI fly ash reduced particle density, whereas increasing the basalt dust content increased it, especially after carbonation in the water glass system. The effect of biomass fly ash was weaker within the studied composition range. The highest particle densities were obtained for basalt-rich mixtures with a low proportion of high-LOI fly ash, while carbonation mainly increased density in the water glass system.

The particle density trends can be explained by the combined effect of precursor density, particle packing, and matrix formation. Basalt dust had the lowest LOI and contained relatively high amounts of SiO_2_, CaO, MgO, Fe_2_O_3_, and TiO_2_, which likely contributed to a more compact granular skeleton and higher particle density. In contrast, the high-LOI fly ash may have increased porosity because thermally removable components, including unburned carbon, can increase liquid demand and reduce the effective formation of a dense binding matrix. The higher density of the Activator 2.5 mixtures, before carbonation, suggests that the addition of NaOH promoted precursor dissolution and early matrix densification. The stronger density increase after carbonation in the water glass system indicates that these initially more open structures were more susceptible to pore filling by carbonate products.

### 3.4. Water Absorption

**Figure 17:**
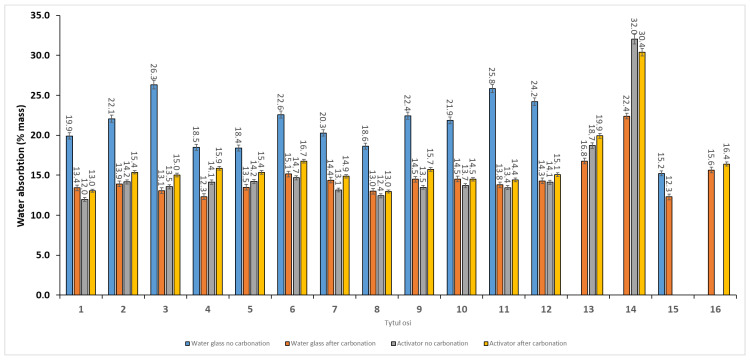
Water absorption of the investigated mixtures (series 1–12) and single material reference series (FA A-100, FA (high-LOI)-100, Bio-100, and Basalt-100) produced using two activator systems (water glass and blended Activator 2.5) and tested before and after carbonation curing. Error bars represent the standard error of the mean (SE).

**Figure 18:**
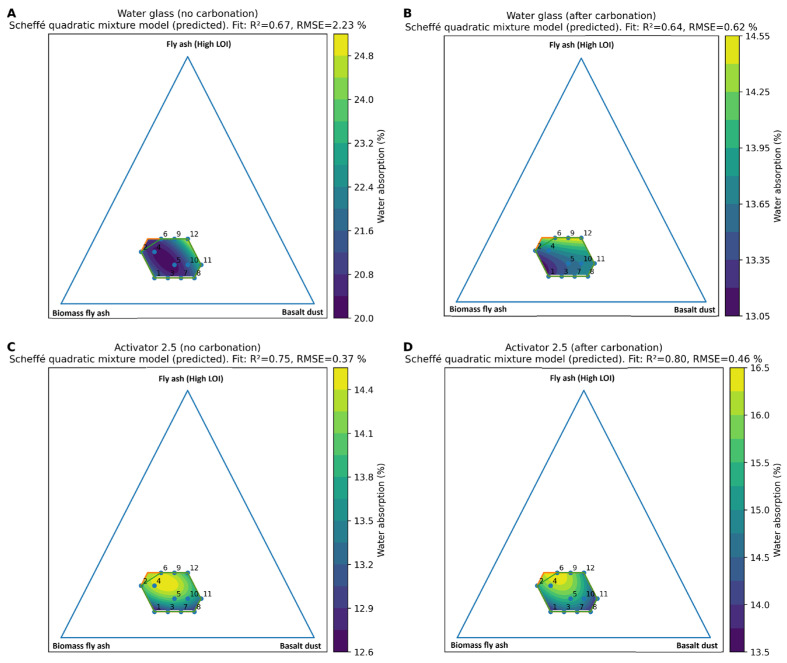
Ternary response–surface plots of water absorption predicted by a Scheffé quadratic mixture model within the constrained design space (Fly Ash A determined at 5 wt.%). (**A**) Water glass, no carbonation; (**B**) water glass, after carbonation; (**C**) Activator 2.5, no carbonation; and (**D**) Activator 2.5, after carbonation.

**Figure 19:**
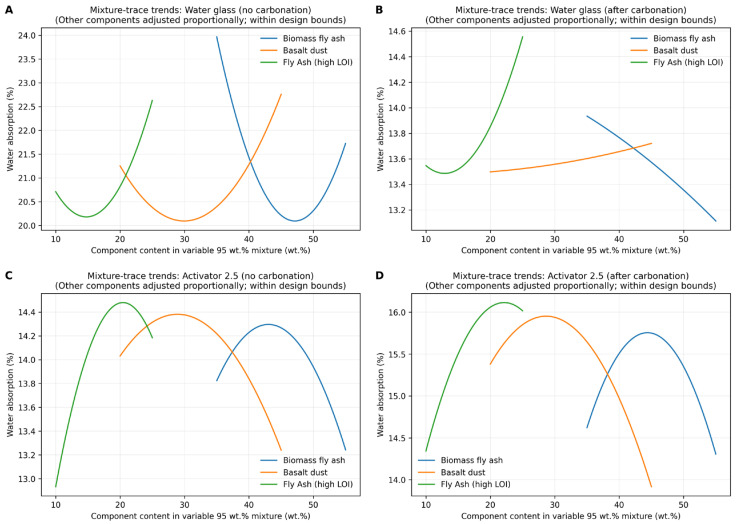
Mixture–trace plots showing the predicted effect of varying each component within the constrained mixture design space (Fly Ash A determined at 5 wt.%). The response is water absorption estimated from a second-order Scheffé mixture model; when one component is varied, the remaining components are adjusted proportionally to maintain the constant total of the variable 95 wt.% fraction. (**A**) Water glass, no carbonation; (**B**) water glass, after carbonation; (**C**) Activator 2.5, no carbonation; and (**D**) Activator 2.5, after carbonation.

Water absorption results for series 1–12 and the 100% reference series are presented in Figure 17, while the composition response relationships are shown in Figure 18 and Figure 19 using Scheffé quadratic mixture models within the constrained design space, with Fly Ash A fixed at 5 wt.%. In the water glass system, the non-carbonated aggregates showed the highest water absorption, ranging from 18.4 to 26.3%. After carbonation, these values decreased markedly to 12.3–15.1%, indicating a reduction in accessible porosity. In contrast, series 8, where the Na_2_SiO_3_/NaOH ratio was 2.5, already exhibited low water absorption before carbonation, with a value of 12.4%, and showed only a limited response to carbonation, reaching 13.0% after CO_2_ exposure. This suggests that the Activator 2.5 system produced a denser initial matrix, whereas the water glass system was more strongly affected by carbonation-induced changes in pore structure. The reference series confirmed the unfavourable effect of high-LOI fly ash, which showed the highest water absorption of approximately 32.0%, compared with FA A and Bio-100, for which the corresponding values were approximately 18.7% and 15.2%, respectively. For the series in which no results are presented, the specimens degraded during conditioning in water, preventing further testing. The fitted models reproduced the main trends with moderate to good agreement (R^2^ ≈ 0.64–0.80; RMSE ≈ 0.37–2.23%). The mixture trace plots indicate that water absorption was governed mainly by the content of high-LOI fly ash, while the effects of biomass fly ash and basalt dust were more moderate and composition-dependent. Overall, basalt-rich mixtures with a low content of high-LOI fly ash, especially series 8, showed the most favourable combination of low water absorption, relatively high density, and good mechanical performance. This behaviour can be attributed to improved particle packing and reduced accessible porosity caused by the fine basalt dust, whereas high-LOI fly ash probably increased liquid demand and hindered the formation of a compact binding matrix.

### 3.5. Optical Microscopy and SEM Observations

The series 8, where the ratio of Na_2_SiO_3_/NaOH = 2.5, was selected for detailed evaluation because it showed the most favourable combination of properties: high single aggregate crushing strength, high density, and low water absorption. The results discussed below refer only to this formulation and should not be considered representative of the entire system.

**Figure 20:**
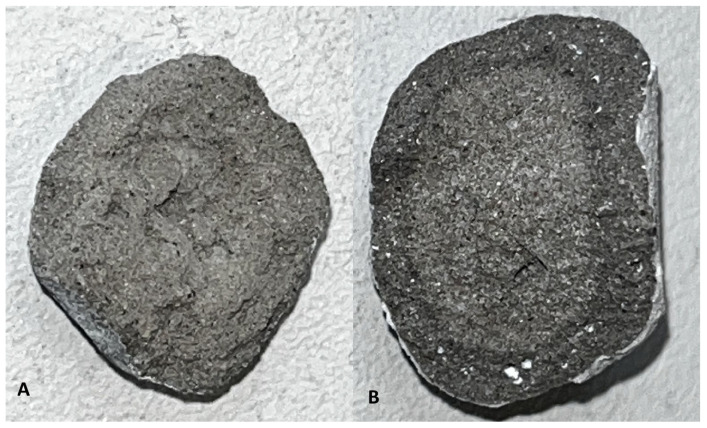
Cross-sections of series 8 specimens prepared with water glass: (**A**) before carbonation and (**B**) after carbonation.

**Figure 21:**
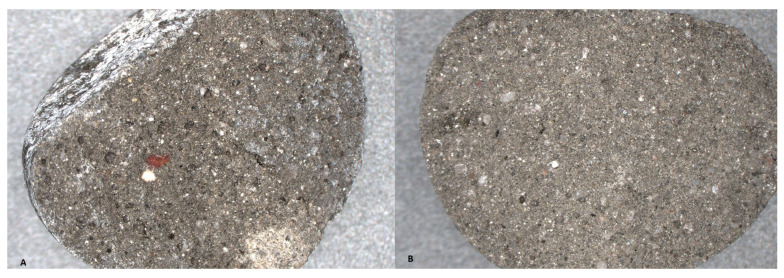
Cross sections of series 8 specimens prepared with Activator 2.5: (**A**) before carbonation and (**B**) after carbonation.

Figure 20 and Figure 21 show representative cross sections of series 8 samples produced with two activator systems before and after carbonation curing. In the water glass series (Figure 20), the carbonated sample appears more compact, with a more distinct outer zone and fewer visible dark pores than the non-carbonated sample. In series 8, where the ratio of Na_2_SiO_3_/NaOH = 2.5, a variant from series 8 was selected for detailed evaluation because, after carbonation, it showed the most favourable combination of properties, namely high single aggregate crushing strength, high density, and low water absorption (Figure 21). The carbonated sample also appears more homogeneous, although the visual differences are less pronounced. These observations are qualitative, but they agree with the measured density and water absorption results, which indicate a stronger densification effect after carbonation in the water glass series and a weaker response in series 8 where ratio of Na_2_SiO_3_/NaOH = 2.5 variant from series 8 was selected for detailed evaluation because, after carbonation, it showed the most favourable combination of properties, namely high single aggregate crushing strength, favourable density, and low water absorption.

**Figure 22:**
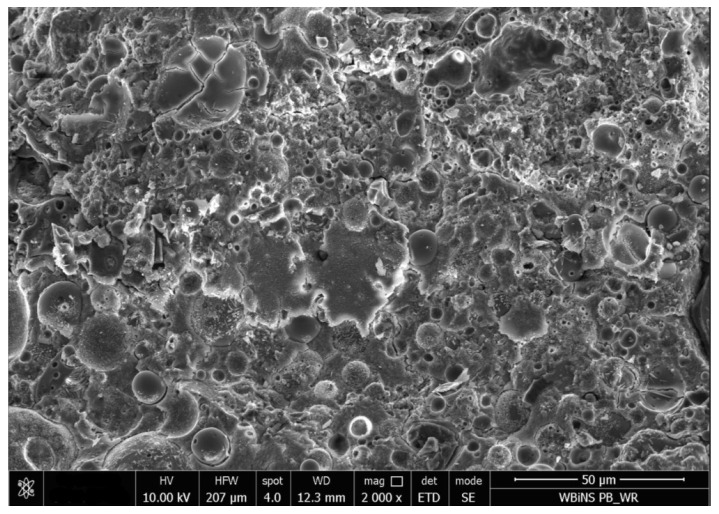
SEM image of the series 8 composite (Activator 2.5) before carbonation, showing an open and heterogeneous matrix with visible pores and microcracks (SE mode, 10 kV; scale bar: 50 µm).

**Figure 23:**
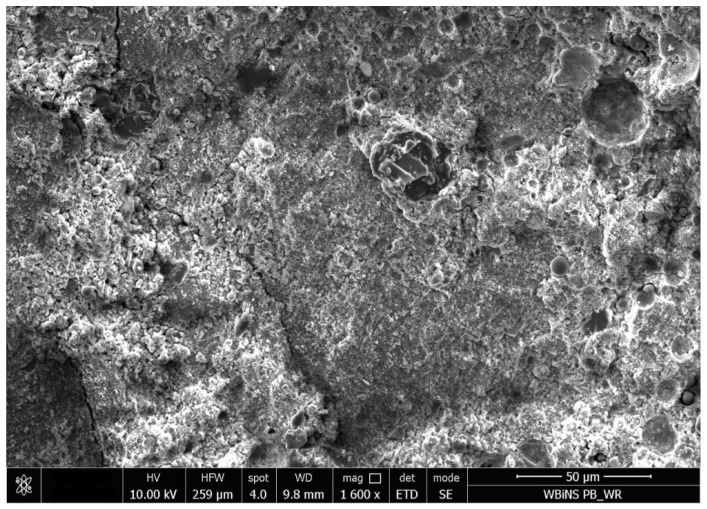
SEM image of the series 8 composite (Activator 2.5) after carbonation, showing a denser and more compact matrix with reduced open porosity and partial pore filling by fine reaction products consistent with carbonation (SE mode, 10 kV; scale bar: 50 µm).

SEM micrographs of the series 8 sample before and after carbonation (Figure 22 and Figure 23) provide qualitative microstructural observations consistent with matrix densification. The uncarbonated sample exhibits a relatively loose and heterogeneous matrix with numerous visible voids and microcracks. After carbonation, the matrix appears more compact and locally more homogeneous, and some voids or interphase gaps appear less open than in the pre-carbonation state. Since no EDS analysis was performed, the fine-grained products visible after carbonation cannot be identified in terms of composition using SEM alone. SEM observations should therefore be considered strictly qualitative and supplementary; nevertheless, they are consistent with the increase in density and decrease in water absorption observed after carbonation in the selected formulation of series 8.

### 3.6. XRD and TG/DTG/DTA Before and After Carbonation

**Figure 24:**
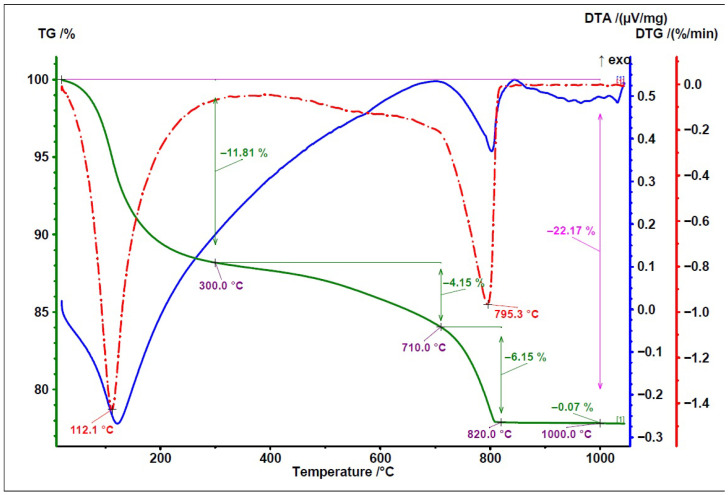
TG/DTG/DTA thermogram of series 8 (Activator 2.5) before carbonation, recorded in air.

**Figure 25:**
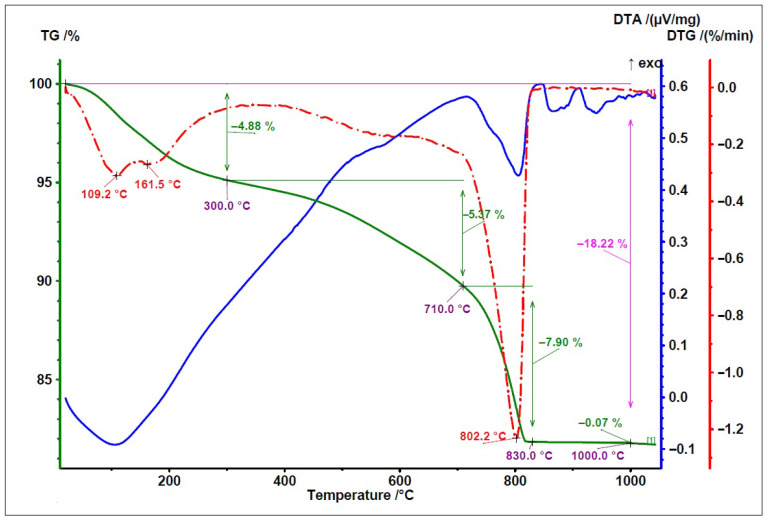
TG/DTG/DTA thermogram of series 8 (Activator 2.5) after carbonation, recorded in air.

The TG/DTG/DTA thermograms recorded in air compare the Series 8 sample prepared with Activator 2.5 before carbonation (Figure 24) and after carbonation (Figure 25). In these figures, the green curve represents TG/mass change, the red dash-dotted curve represents DTG, and the blue curve represents DTA; the additional guide lines indicate selected temperature ranges and corresponding mass-loss values.In both cases, three main regions of mass change can be identified: (i) low temperature mass loss, mainly related to the evaporation of free and physically bound water and the dehydration of weakly bound phases; (ii) gradual mass loss in the range of about 300–710 °C, associated with the dehydration; (iii) a sharp mass loss step between about 710 and 820–830 °C, attributed mainly to the decomposition of carbonates. The greater magnitude of this high-temperature mass loss after carbonation (−7.90% vs. −6.15%) indicates a higher carbonate content in the carbonated composite.

A preliminary estimate of CO_2_ uptake was calculated from the difference in mass loss within the selected decarbonation range between the carbonated and non-carbonated series 8 samples, if this difference mainly reflects the additional decomposition of carbonates formed during carbonation curing. On this basis, the CO_2_ uptake was estimated at 17.5 g CO_2_/kg. This value should be treated as approximate and specific to this formulation, because the selected thermal range may include overlapping contributions from different carbonate phases, and no independent phase quantification was performed.

**Figure 26:**
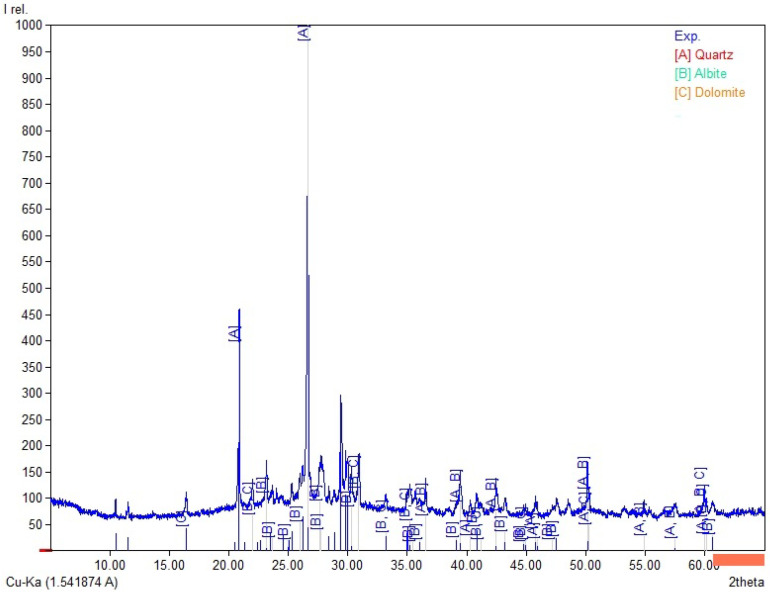
XRD pattern of the series 8 composite (Activator 2.5) before carbonation, showing the dominant crystalline phases: quartz (A), albite (B), and dolomite (C).

**Figure 27:**
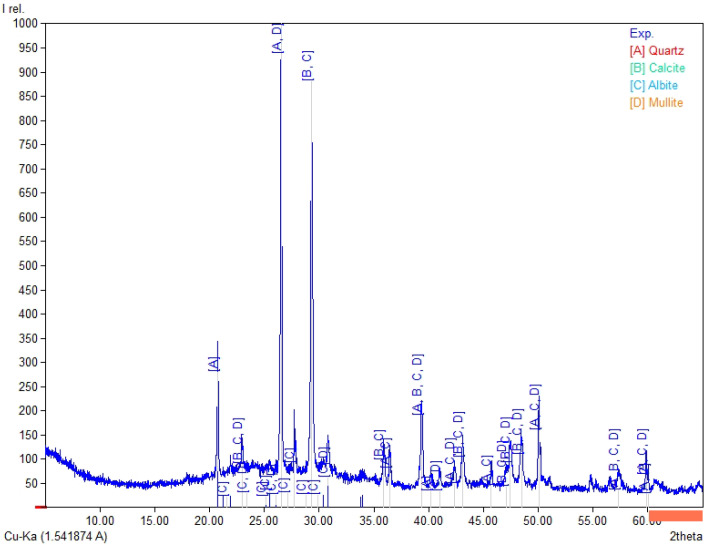
XRD pattern of the series 8 composite (Activator 2.5) after carbonation, showing quartz (A) and albite (C) with the appearance/intensification of calcite (B; CaCO_3_) and minor mullite (D), confirming mineral carbonation (CO_2_ sequestration).

The XRD patterns of the selected series 8 composites (Activator 2.5 series) before and after carbonation (Figure 26 and Figure 27) show phase changes related to carbonation and contain crystalline phases inherited from the precursors.

In the non-carbonated sample (Figure 26), the main crystalline phases are quartz (A), albite (B), and dolomite (C). After carbonation (Figure 27), quartz and albite remain present, while the calcite peaks become more pronounced, suggesting additional carbonate formation during CO_2_ exposure. Minor mullite is also detected in the carbonated sample. No quantitative phase analysis was performed, and the amorphous fraction was not determined; therefore, the XRD results should be treated as qualitative only. Together with the larger carbonate-related mass loss observed in the TG/DTG curves, these results support the interpretation that Ca-bearing components in the selected formulation underwent carbonation and that CO_2_ absorption occurred during carbonation curing.

The thermal analysis (TG/DTG) and XRD analysis results are consistent with the physical-mechanical properties trends observed for series 8. The increase in carbonate-related weight loss following carbonation and the enhanced calcite reflection indicate that exposure to CO_2_ promoted additional carbonate formation in the selected formulation. As a result, carbonate phases may have contributed to partial pore filling and local matrix densification, which corresponds with the SEM observations and high single aggregate crushing strength of series 8 after carbonation.

### 3.7. Screening Carbon Footprint Assessment

A screening cradle-to-gate carbon footprint (A1–A3) was estimated for series 8 produced with Activator 2.5 (Table 4). Under the adopted assumptions, the calculated value was 0.256 kg CO_2_ eq/kg of dry precursor mixture, i.e., 256 kg CO_2_ eq/t. This value is markedly higher than those reported in Polish EPDs for natural aggregates, for which A1–A3 values of 3.28 kg CO_2_ eq/t for natural crushed aggregate [37] and 3.99 kg CO_2_ eq/t for basalt aggregate have been reported [38]. At the same time, the obtained result is close to that reported for a fired expanded clay lightweight aggregate. For ARGEX AR 8/16–340 GEO, the EPD according to EN 15804+A2 reports a GWP total of 10.15 kg CO_2_ eq for a functional unit of 38.76 kg, which corresponds to approximately 262 kg CO_2_ eq/t [39]. Thus, the present screening estimate for series 8 is substantially higher than for natural aggregates, but broadly comparable to this specific expanded clay product. In addition, TG/DTG analysis of carbonated series 8 indicated a preliminary CO_2_ uptake of about 17.5 g CO_2_/kg. When this approximate value is considered only as a sensitivity case, the net result decreases to about 238.5 kg CO_2_ eq/t. However, this uptake estimate should be treated as formulation-specific and approximate, since it was derived from differential mass loss in the selected decarbonation range without independent quantitative phase determination.

## 4. Conclusions

The results show that biomass fly ash, coal fly ash, and basalt dust can be used to produce alkali-activated granular aggregates with adjustable properties. The mixture of sodium silicate and NaOH (Activator 2.5) was generally more advantageous than sodium silicate alone, and mixtures with a high basalt content and low LOI value provided the best overall performance. Series 8 exhibited one of the most favourable combinations of properties, with a single aggregate crushing strength after carbonation of 8.3–8.4 MPa, as well as high density (1.63–1.91 g/cm^3^) and low water absorption (12.4–18.6%). Changes associated with carbonation were confirmed by TG/DTG, XRD, and SEM observations, and the initial CO_2_ absorption for series 8 was estimated at 17.5 g CO_2_/kg. The carbon footprint of A1–A3 for this formulation was 256 kg CO_2_ equivalent/t, decreasing to approximately 238.5 kg CO_2_ equivalent/t when the initial CO_2_ absorption was considered a sensitivity case. Overall, the proposed method represents a viable approach to the valorization of industrial mineral waste, although it is still necessary to reduce the load on the activator system.

## Figures and Tables

**Table 1:** Sodium silicate solution and sodium hydroxide specifications.

Material	Sodium Silicate Solution (Water Glass)
Nominal composition	Na_2_O·nSiO_2_·nH_2_O
CAS number	1344-09-8
Supplier	CHEMPUR (Piekary Slaskie, Poland)
Na_2_O content (wt.%)	11.1
SiO_2_ content (wt.%)	27.9
Total solids (wt.%)	~39.0
Density at 20 °C (g·cm^−3^)	1.47
Silicate modulus (SiO_2_/Na_2_O, mass ratio)	2.51
Impurities (wt.%)	Fe_2_O_3_ 0.01; CaO 0.1; insoluble matter 0.1

**Table 2:** Mix design of raw-material blends (wt.%), including single-material reference mixture.

Series	Biomass Fly Ash [%]	Basalt Dust [%]	Fly Ash (High-LOI) [%]	Fly Ash A [%]
1	55	30	10	5
2	55	20	20	5
3	50	35	10	5
4	50	25	20	5
5	45	35	15	5
6	45	25	25	5
7	45	40	10	5
8	40	45	10	5
9	40	30	25	5
10	40	40	15	5
11	35	45	15	5
12	35	35	25	5
13—FA A-100	0	0	0	100
14—FA (high-LOI)-100	0	0	100	0
15—Bio-100	100	0	0	0
16—Basalt-100	0	100	0	0

**Table 3:** The chemical composition of fly ashes and basalt dust is used to produce artificial aggregate.

	Fly Ash A	Biomass Fly Ash	Fly Ash—High-LOI	Basalt Dust
	[%]			
SiO_2_	38.583	29.711	35.535	37.183
Al_2_O_3_	16.146	7.548	15.1	10.179
Fe_2_O_3_	5.505	3.834	6.023	12.789
CaO	2.142	16.033	1.878	8.744
MgO	0.843	1.999	0.292	5.923
K_2_O	2.944	3.636	1.924	1.277
Na_2_O	-	-	1.421	-
TiO_2_	1.189	0.65	1.19	2.526
P_2_O_5_	0.483	1.655	1.104	0.677
SO_3_	0.485	2.322	0.745	0
Cl	-	0.21	-	0.051
MnO	0.047	0.564	0.036	0.188
Cr_2_O_3_	0.028	0.011	0.025	0.026
V_2_O_5_	0.053	0.016	0.065	0.044
NiO	0.019	0.00349	0.027	0.022
CuO	0.018	0.00943	0.021	0.00755
ZnO	0.024	0.054	0.02	0.015
BaO	0.078	0.055	0.077	0.036
SrO	0.08	0.034	0.163	0.1
LOI	4.5	9.1	10.8	3.58

**Table 4:** Screening cradle to gate carbon footprint (A1–A3) of series 8 (Activator 2.5), calculated per 1 kg of dry precursor mixture.

Module	Input/Process	Quantity [kg]	Emission Factor [kg CO_2_ eq/kg]	GWP (kg CO_2_ eq)
A1	Biomass fly ash	0.4000	0.000	0.00000
A1	Basalt dust	0.4500	0.000	0.00000
A1	Fly ash (high-LOI)	0.1000	0.000	0.00000
A1	Fly Ash A	0.0500	0.000	0.00000
A1	Sodium silicate solution	0.2381	0.554	0.13190
A1	Pure NaOH used to prepare 12 M solution	0.0333	1.676	0.05587
A1	Water used for NaOH solution preparation	0.0619	excluded	0.00000
	Subtotal A1			0.18777
A2	Transport of biomass fly ash (6 km)	0.4000	0.146 kg CO_2_ eq/t·km	0.00035
A2	Transport of basalt dust (400 km)	0.4500	0.146 kg CO_2_ eq/t·km	0.02628
A2	Transport of fly ash (high-LOI) (6 km)	0.1000	0.146 kg CO_2_ eq/t·km	0.00009
A2	Transport of Fly Ash A (118 km)	0.0500	0.146 kg CO_2_ eq/t·km	0.00086
A2	Transport of sodium silicate solution (500 km)	0.2381	0.146 kg CO_2_ eq/t·km	0.01738
A2	Transport of pure NaOH (500 km)	0.0333	0.146 kg CO_2_ eq/t·km	0.00243
A2	Transport of water for NaOH solution preparation	local	excluded	0.00000
	Subtotal A2			0.04739
A3	Granulation	0.03333 kWh	0.617 kg CO_2_ eq/kWh	0.02057
	Subtotal A3			0.02057
	Total A1–A3			0.25573

## Data Availability

The original contributions presented in this study are included in the article. Further inquiries can be directed to the corresponding author.
